# Weight-Bearing Physical Activity, Lower-Limb Muscle Mass, and Risk of Knee Osteoarthritis

**DOI:** 10.1001/jamanetworkopen.2024.8968

**Published:** 2024-04-30

**Authors:** Yahong Wu, Cindy G. Boer, Amy Hofman, Dieuwke Schiphof, Marienke van Middelkoop, Ingrid A. Szilagyi, Bahar Sedaghati-Khayat, Sita M. A. Bierma-Zeinstra, Trudy Voortman, Joyce B. J. van Meurs

**Affiliations:** 1 Department of Epidemiology, Erasmus Medical Center, University Medical Center Rotterdam, Rotterdam, the Netherlands; 2 Department of Internal Medicine, Erasmus Medical Center, University Medical Center Rotterdam, Rotterdam, the Netherlands; 3 Department of General Practice, Erasmus Medical Center, University Medical Center Rotterdam, Rotterdam, the Netherlands; 4 Department of Orthopedics & Sports Medicine, Erasmus Medical Center, University Medical Center Rotterdam, Rotterdam, the Netherlands

## Abstract

**Importance:**

It has been demonstrated that total physical activity is not associated with risk of osteoarthritis. However, the association of different types of physical activity with incident knee osteoarthritis remains unclear.

**Objective:**

To determine whether weight-bearing recreational physical activities are associated with increased risk of incident knee osteoarthritis.

**Design, Setting, and Participants:**

This prospective cohort study used data from the Rotterdam Study (1996 to 2009), including participants with knee x-ray measurements at baseline and follow-up examinations. Participants with knee osteoarthritis at baseline were excluded. Residents aged 45 years and older of the Ommoord district in the city of Rotterdam in The Netherlands were invited to join the Rotterdam Study (78% response rate). Analysis was conducted in June 2023.

**Exposure:**

Total, weight-bearing, and non–weight-bearing recreational physical activities collected by questionnaires at baseline.

**Main Outcomes and Measures:**

Incident radiographic knee osteoarthritis measured by knee x-ray was the primary outcome, and incident symptomatic knee osteoarthritis defined by x-ray and knee pain questionnaire was the secondary outcome. The association of different types of recreational physical activity with radiographic knee osteoarthritis was examined using logistic regression within generalized estimating equation framework after adjusting for potential confounders. A prespecified stratification analysis was planned on the basis of lower-limb muscle mass index (LMI) tertiles, measured by dual-energy x-ray absorptiometry.

**Results:**

A total of 5003 individuals (2804 women [56.0%]; mean [SD] age, 64.5 [7.9] years) were included. The knee osteoarthritis incident rate was 8.4% (793 of 9483 knees) for a mean (SD) follow-up time of 6.33 (2.46) years. Higher weight-bearing activity was associated with increased odds of incident knee osteoarthritis (odds ratio [OR], 1.22; 95% CI, 1.10-1.35; *P* < .001), but non–weight-bearing activity was not (OR, 1.04; 95% CI, 0.95-1.15; *P* = .37). In the analysis stratified by LMI tertiles, the association of weight-bearing activity with incident osteoarthritis was found only among 431 patients in the lowest LMI tertile (OR, 1.53; 95% CI, 1.15-2.04; *P* = .003), but not among patients in the middle or high LMI tertile.

**Conclusions and Relevance:**

The findings of this study suggest that weight-bearing activity is associated with incident knee osteoarthritis in people with low levels of lower-limb muscle mass, which might be a promising avenue for tailored advice for physical activity.

## Key Points

**Question:**

Are weight-bearing recreational physical activities associated with increased risk of knee osteoarthritis?

**Findings:**

In this cohort study of 5003 participants, weight-bearing recreational physical activity was significantly associated with increased odds of incident knee osteoarthritis among participants with low levels of lower-limb muscle mass.

**Meaning:**

This study provides evidence for future tailored physical activity recommendations based on a person’s muscle mass and osteoarthritis risk, which can help optimize the benefits of physical activity while minimizing the potential risk of developing osteoarthritis.

## Teaser

This cohort study examines whether weight-bearing recreational physical activities are associated with incident knee osteoarthritis.

## Introduction

Osteoarthritis is a degenerative joint disease characterized by chronic pain and limited joint movement, leading to substantial health burden and socioeconomic costs owing to its high prevalence.^1,2,3^ Currently, there is no disease-modifying treatment for osteoarthritis. Thus, developing effective prevention strategies and identifying modifiable risk factors are essential. One possible factor associated with the risk of osteoarthritis is physical activity.^4^ However, in the past, conflicting results were found regarding the effect of physical activity on knee osteoarthritis.^4,5,6,7,8^ Recently, a large individual participant–level data meta-analysis^8^ across 6 cohorts of 5065 individuals set out to elucidate whether physical activity was associated with increased risk of knee osteoarthritis. That study found no association of total physical activity with increased risk for knee osteoarthritis.^8^

Physical activity, as a potential risk factor for osteoarthritis, was suggested to act through the loading force on the joint during the physical activity. That recent large individual participant–level data meta-analysis^8^ found no increased risk of osteoarthritis when examining the total physical activity. However, different types of physical activity impose varying loads on the knees. For example, a weight-bearing activity like running can subject the knee to an impact as high as twice the individual’s body weight. In contrast, non–weight-bearing activities like swimming do not have this same effect.^9^ It may be possible that the effect of physical activity on knee osteoarthritis varies depending on the type of physical activity. Unfortunately, the large meta-analysis study could not investigate the different types of physical activity with the risk of osteoarthritis.^8^

In addition, the risk of osteoarthritis is mediated by not only the diverse loading forces associated with different types of physical activity but also by how an individual’s joint can cope with such loading forces. The muscles surrounding the joint play a critical role in physical activity: lower-limb muscles are the main components in keeping the knee joint stable during activity,^10,11^ and they can act like a cushion, absorbing loading force during activity.^12^ Not surprisingly, lower-limb muscle weakness has also been identified as a factor associated with the risk of osteoarthritis.^12,13,14^ Thus, it may be possible that the effect of physical activity on knee osteoarthritis depends on the muscle surrounding the knee joint. If this is the case, discovering specific conditions under which physical activity acts as a risk or protective factor for osteoarthritis would benefit patients and health care practitioners.

To address this research gap, we aimed to investigate (1) the association of weight-bearing and non–weight-bearing recreational physical activity with the risk of incident knee osteoarthritis and (2) whether this association is mediated by the lower-limb muscle mass. This study is embedded in the Rotterdam Study (RS), a large and deeply phenotyped prospective population cohort.^15^ We used lower-limb muscle mass measured by dual x-ray absorptiometry (DXA) to represent muscle strength because the strength data are not available in RS.^16^

## Methods

### Study Design and Population

**Figure 1.**
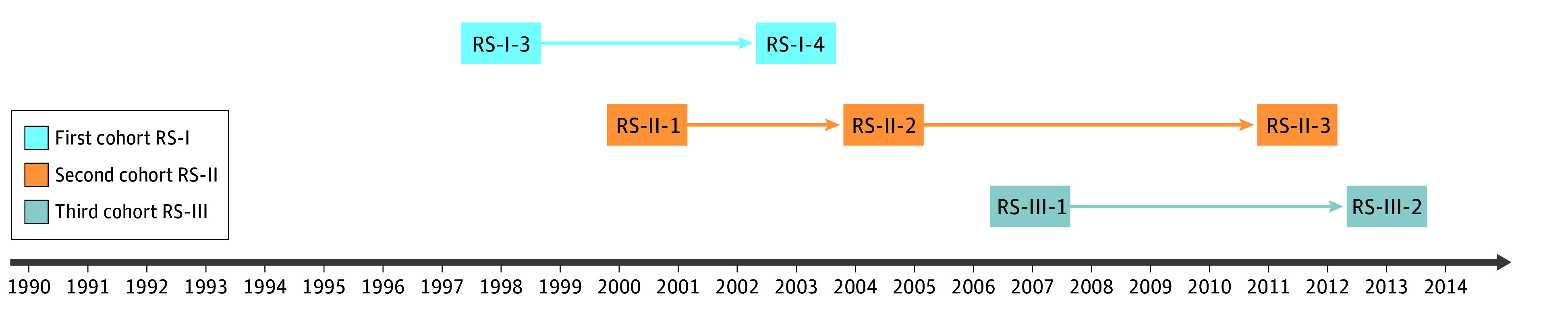
Design of the Rotterdam Study (RS) RS-I-3 and RS-I-4 refer to re-examinations of the original cohort members. RS-II-1 refers to the extension of the cohort with persons from the study district who had turned 55 years old since the start of the study or those aged 55 years or older who migrated into the study district. RS-II-2 and RS-II-3 refer to re-examinations of the extension cohort. RS-III-1 refers to another extension of the cohort with persons aged 45 years and older living in the study district who had not been examined already (ie, mainly comprising those aged 45-60 years). RS-III-2 refers to the first re-examination of this third cohort.

This study was embedded in the RS, a large population-based prospective cohort study started in 1990. The design of the RS has been previously described in detail^15^ (Figure 1 and eAppendix in Supplement 1). The RS has been approved by the Medical Ethics Committee of the Erasmus Medical Center and by the Dutch Ministry of Health, Welfare and Sport (Population Screening Act WBO). Written informed consent was obtained from all participants. This study follows the Strengthening the Reporting of Observational Studies in Epidemiology (STROBE) reporting guidelines.

**Figure 2.**
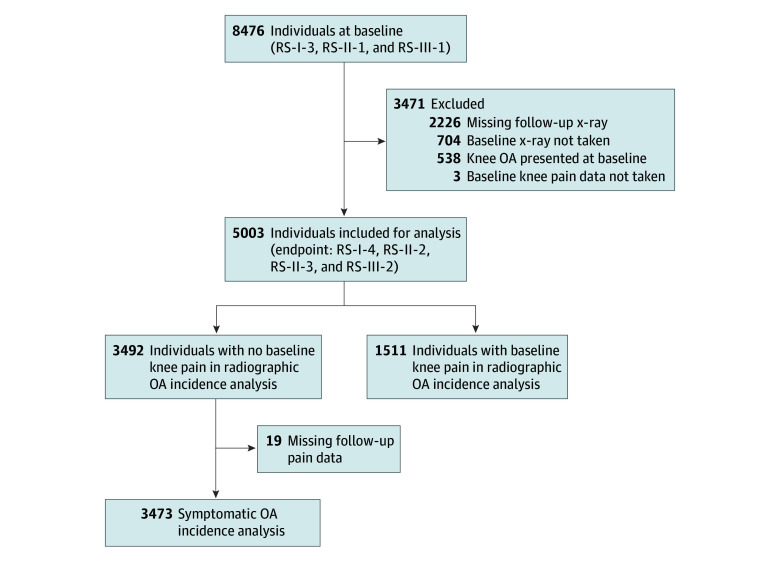
Flowchart for Individuals Included for Analysis Chart shows overview of the Rotterdam Study (RS) population and performed analysis. Radiographic knee osteoarthritis (OA) incidence was defined as baseline knee Kellgren and Lawrence grade of 1 or lower and 2 or higher at follow-up or having a total knee replacement at follow-up. Symptomatic OA incidence was defined as baseline knee Kellgren and Lawrence grade 1 or lower with negative knee pain, combined with Kellgren and Lawrence grade 2 or higher, or having a total knee replacement at follow-up with positive knee pain.

The current analysis included participants from the 3 RS subcohorts (RS-I, RS-II, and RS-III) who had complete data of baseline recreational physical activity, baseline knee pain, and knee radiographs from baseline and at least 1 time from follow-up visits. Participants with x-ray–defined osteoarthritis for 1 or both knees at baseline were excluded (Figure 2). More details about the study population are shown in the eAppendix in Supplement 1.

### Recreational Physical Activity

Total, weight-bearing, and non–weight-bearing recreational physical activity data were collected using validated questionnaires, an adapted version of the Zutphen Physical Activity Questionnaire, and the Longitudinal Aging Study Amsterdam physical activity questionnaire at baseline visit,^17,18^ expressed in metabolic equivalent of task (MET).^19^ Classification of weight-bearing and non–weight-bearing physical activity levels was based on data from a previous biomechanical study^9^ (eAppendix, eTable 1, and eTable 2 in Supplement 1).

### Osteoarthritis

We assessed 2 outcomes: incident radiographic knee osteoarthritis based on x-ray radiographs and incident symptomatic knee osteoarthritis based on a self-reported pain questionnaire and x-ray radiographs. Radiographic knee osteoarthritis is the primary outcome, and symptomatic knee osteoarthritis is the secondary outcome (eAppendix in Supplement 1).

### Measurements of Lower-Limb Muscle Mass

Lower-limb muscle mass was measured by DXA (Prodigy and iDXA devices, GE Healthcare),^20,21^ in a subgroup of subcohorts RS-II and RS-III. To adjust for height, we calculate a lower-limb muscle mass index (LMI) as lower limb lean mass in kilograms divided by height in meters squared.^16^

### Covariates

We used the following covariates: age, sex, body mass index (calculated as weight in kilograms divided by height in meters squared), baseline Kellgren and Lawrence osteoarthritis grade (KLG; 0, none; 1, doubtful; 2, minimal; 3, moderate; and 4, severe), RS subcohorts, education level, alcohol intake, smoking, systolic blood pressure, ratio of high-density lipoprotein to total cholesterol ratio, and diabetes prevalence. Details are shown in the eAppendix in Supplement 1.

### Statistical Analysis

Data analysis was conducted in June 2023. The association of recreational physical activity at baseline (total, weight-bearing, and non–weight-bearing) with incidence of radiographic and symptomatic osteoarthritis was assessed using logistic regression (statistical significance threshold, 2-sided *P* < .05), adjusted for RS subcohorts, baseline KLG, follow-up time, age, sex, and body mass index (model 1). When analyzing incident radiographic knee osteoarthritis, both knees from the same individual were included. A generalized estimating equation was used to account for the correlation of the knees from the same participant. We also adjusted for education level, alcohol intake, smoking, systolic blood pressure, ratio of high-density lipoprotein cholesterol to total cholesterol, and diabetes prevalence (model 2). To study whether LMI mediates the association of recreational physical activity with osteoarthritis, a prespecified stratification analysis based on sex-specific tertiles of LMI was conducted in the subgroup of participants with LMI data available. To reduce the chance of type I error, Benjamin-Hochberg multiple testing correction was applied to all analysis results with a false discovery rate of 0.05.^22^ All analyses were done separately in participants with and without baseline pain (eAppendix in Supplement 1). Data were analyzed using R statistical software version 4.2.1 (R Project for Statistical Computing).

## Results

### Baseline Characteristics

This study used data from 3 RS subcohorts (RS-I, RS-II, and RS-III) for 5003 individuals (2804 women [56.0%]; mean [SD] age, 64.5 [7.9] years) who had complete data of baseline recreational physical activity, baseline knee pain, and knee radiographs at both baseline and follow-up visits (Table 1). We noted differences in baseline characteristics between the study population and those lost during follow-up (2224 patients) (eTable 3 in Supplement 1). The knee osteoarthritis incident rate was 8.4% (793 of 9483 knees) for a mean (SD) follow-up time of 6.33 (2.46) years. At baseline, no participants had knee osteoarthritis, 1587 participants (31.7%) had a KLG of 1, and 1511 participants (30.2%) reported knee pain. The mean (SD) total recreational physical activity was 43.6 (33.6) MET hours per week, of which 76% (mean [SD], 32.4 [27.7] MET hours per week) was weight-bearing activity and 24% (mean [SD], 11.2 [15.8] MET hours per week) was non–weight-bearing activity (Table 1). In weight-bearing activity, 89% was contributed by daily activity like walking, whereas the remaining 11% was from regular sports such as running. In non–weight-bearing activity, 86% came from a daily activity like biking, whereas the remaining 14% came from regular sports participation such as swimming (eTable 4 in Supplement 1).

**Table 1.** Baseline Characteristics of the Study Population

Characteristic	Patients, No (%)						
	Total (N = 5003)	Without baseline knee pain			With baseline knee pain		
		Total (n = 3492)	Men (n = 1684)	Women (n = 1808)	Total (n = 1511)	Men (n = 515)	Women (n = 996)
Sex							
Men	2199 (44.0)	1684 (48.2)	1684 (100.0)	0	515 (34.1)	515 (100.0)	0
Women	2804 (56.0)	1808 (51.8)	0	1808 (100.00)	996 (65.9)	0	996 (100.0)
Age, mean (SD), y	64.48 (7.89)	64.75 (7.97)	64.88 (7.83)	64.63 (8.11)	63.86 (7.67)	64.14 (7.43)	63.71 (7.78)
RS subcohorts							
RS-I	2172 (43.4)	1642 (47.0)	812 (48.2)	830 (45.9)	530 (35.1)	180 (35.0)	350 (35.1)
RS-II	1470 (29.4)	912 (26.1)	457 (27.1)	455 (25.2)	558 (36.9)	203 (39.4)	355 (35.6)
RS-III	1361 (27.2)	938 (26.9)	415 (24.6)	523 (28.9)	423 (28.0)	132 (25.6)	291 (29.2)
Follow-up time, mean (SD), y	6.33 (2.46)	6.15 (2.38)	6.19 (2.44)	6.12 (2.32)	6.73 (2.61)	6.81 (2.70)	6.69 (2.56)
Body mass index, mean (SD)^a^	26.95 (3.92)	26.65 (3.72)	26.64 (3.28)	26.65 (4.09)	27.64 (4.27)	27.59 (3.50)	27.67 (4.61)
Lower limb muscle index, mean (SD), kg/m^2^	5.57 (0.85)	5.60 (0.86)	6.30 (0.61)	5.03 (0.56)	5.52 (0.81)	6.36 (0.64)	5.10 (0.51)
Education level							
Primary education	502 (10.1)	326 (9.4)	116 (6.9)	210 (11.7)	176 (11.7)	45 (8.8)	131 (13.3)
Lower or intermediate general or lower vocational education	2076 (41.8)	1419 (40.9)	475 (28.4)	944 (52.6)	657 (43.8)	143 (27.9)	514 (52.0)
Intermediate vocational or higher general education	1483 (29.8)	1064 (30.7)	625 (37.3)	439 (24.5)	419 (27.9)	203 (39.6)	216 (21.9)
Higher vocational education or university	908 (18.3)	660 (19.0)	459 (27.4)	201 (11.2)	248 (16.5)	121 (23.6)	127 (12.9)
Smoking							
Never smoker	2064 (41.3)	1360 (39.0)	434 (25.8)	926 (51.2)	704 (46.6)	153 (29.7)	551 (55.3)
Former smoker	2335 (46.7)	1673 (47.9)	1043 (61.9)	630 (34.9)	662 (43.8)	312 (60.6)	350 (35.1)
Current smoker	603 (12.1)	458 (13.1)	207 (12.3)	251 (13.9)	145 (9.6)	50 (9.7)	95 (9.5)
Alcohol intake, mean (SD), g/d	11.19 (14.16)	11.25 (13.49)	15.03 (15.52)	7.73 (10.06)	11.03 (15.65)	17.62 (21.22)	7.70 (10.40)
Ratio of high-density lipoprotein cholesterol to total cholesterol, mean (SD)	0.25 (0.08)	0.25 (0.08)	0.23 (0.07)	0.27 (0.08)	0.25 (0.08)	0.23 (0.07)	0.26 (0.08)
Systolic blood pressure, mean (SD), mm Hg	139 (21)	140 (21)	141 (20)	138 (21)	138 (20)	140 (19)	137 (21)
Diabetes at baseline	497 (9.9)	351 (10.1)	204 (12.1)	147 (8.1)	146 (9.7)	66 (12.8)	80 (8.0)
Baseline Kellgren and Lawrence grade of 1	1587 (31.7)	1024 (29.3)	460 (27.3)	564 (31.2)	563 (37.3)	179 (34.8)	384 (38.6)
Total physical activity, mean (SD), MET hr/wk	43.6 (33.6)	44.5 (34.5)	46.7 (36.2)	42.5 (32.8)	41.6 (31.1)	43.2 (33.7)	40.7 (29.6)
Weight-bearing physical activity, mean (SD), MET hr/wk	32.4 (27.7)	33.5 (28.7)	34.2 (29.2)	32.8 (28.3)	29.94 (24.9)	29.5 (25.0)	30.2 (24.9)
Non–weight-bearing physical activity, mean (SD), MET hr/wk	11.2 (15.8)	11.0 (15.7)	12.4 (17.2)	9.71 (13.99)	11.6 (16.2)	13.7 (19.4)	10.5 (14.2)

Abbreviations: MET, metabolic equivalent of task; RS, Rotterdam Study.

^a^ Body mass index is calculated as weight in kilograms divided by height in meters squared.

### Association of Recreational Physical Activity With Incident Knee Osteoarthritis

First, we examined the association of different types of recreational physical activity, both weight-bearing and non–weight-bearing, with incident knee osteoarthritis. Because knee pain may influence the amount of recreational physical activity an individual performs,^23^ we excluded 1511 participants with knee pain at baseline. We found no increased odds of incident radiographic osteoarthritis with non–weight-bearing activity (odds ratio [OR], 1.04; 95% CI, 0.95-1.15; *P* = .37) (Table 2). In comparison, we saw a significant association of weight-bearing activity with radiographic osteoarthritis incidence (OR, 1.22; 95% CI, 1.10-1.35; *P* < .001) (Table 2). These results remained the same, even if we also adjusted for more confounders (model 2) (Table 2). However, we did not identify any association of the incidence of symptomatic knee osteoarthritis with total physical activity, weight-bearing activity, or non–weight-bearing activity (3473 participants) (eTable 5 in Supplement 1).

**Table 2.** Association of Recreational Physical Activity With Incident Knee Radiographic Osteoarthritis in Population Without Baseline Knee Pain^a^

Exposure	Unadjusted model^b^		Model 1^b^		Model 2^b^	
	OR (95% CI)	*P* value	OR (95% CI)	*P* value	OR (95% CI)	*P* value
Total recreational physical activity	1.13 (1.02-1.25)	.02^c^	1.21 (1.08-1.34)	.001^c^	1.20 (1.07-1.34)	.001^c^
Non–weight-bearing activity	0.99 (0.89-1.1)	.83	1.04 (0.95-1.15)	.37	1.04 (0.94-1.14)	.44
Weight-bearing activity	1.16 (1.05-1.29)	.003^c^	1.22 (1.10-1.35)	<.001^c^	1.21 (1.09-1.35)	<.001^c^

Abbreviation: OR, odds ratio.

^a^ Of 3492 patients with 6725 knee joints, 415 joints (6.17%) had radiographic evidence of osteoarthritis.

^b^ The statistical model used is a generalized estimating equation multivariate logistic regression model. Model 1 was adjusted for age, sex, Rotterdam Study subcohorts, body mass index, follow-up time, and baseline Kellgren and Lawrence grade. Model 2 additionally was adjusted for education level, alcohol intake, smoking, systolic blood pressure, ratio of high-density lipoprotein cholesterol to total cholesterol ratio, and diabetes.

^c^ Indicates *P* value remain significant after multiple testing corrections using Benjamini and Hochberg method.

To see whether baseline pain mediates the association of recreational physical activity with osteoarthritis, we also repeated these analyses in 1511 individuals with baseline knee pain. We observed no significant association of recreational physical activity levels (total, weight-bearing, or non–weight-bearing) with incident radiographic knee osteoarthritis (eTable 6 in Supplement 1). However, estimated ORs were similar to those found in the population without knee pain (Table 2).

### Associations of Recreational Physical Activity With Incident Knee Osteoarthritis in Individuals With Different LMI

**Figure 3.**
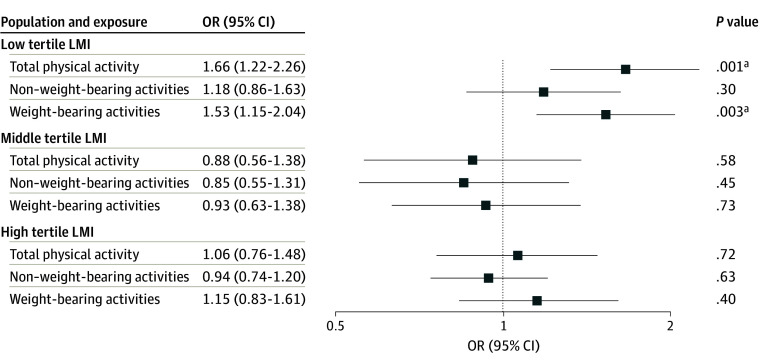
Association of Recreational Physical Activity and Osteoarthritis Stratified by Lower Limb Muscle Index (LMI) Tertiles The statistical model used is a generalized estimating equation multivariate logistic regression model. Results are from the model adjusted for age, sex, Rotterdam Study subcohorts, body mass index, follow-up time, and baseline Kellgren and Lawrence grade. OR indicates odds ratio. ^a^Indicates that *P* value remained significant after multiple testing corrections using Benjamini and Hochberg method.

Because the muscles surrounding the joint play a critical role in the effect of physical activity on the joint,^10,11^ we also investigated the association of physical activity with osteoarthritis in 1881 individuals with different LMI measured by DXA scan. We noted differences in baseline characteristics between the total population and this subgroup (eTable 7 in Supplement 1). We stratified our population into LMI-based tertiles (eTable 8 in Supplement 1). In 1273 individuals free of baseline knee pain, we observed a significant association of weight-bearing activity with incident radiographic knee osteoarthritis among 431 patients in the lowest LMI tertile after multiple testing correction (model 1, OR, 1.53; 95% CI, 1.15-2.04; *P* = .003) but not among patients in the middle and high LMI tertile (Figure 3 and eTable 9 in Supplement 1). This association remained stable after additionally adjusting for more confounders but lost statistical significance after multiple testing corrections (model 2, OR, 1.52; 95% CI, 1.08-2.14; *P* = .02; corrected *P* = .09) (eTable 9 in Supplement 1). Regarding non–weight-bearing activity, no significant association with the odds of osteoarthritis was found across all LMI tertiles (Figure 3 and eTable 9 in Supplement 1). For 608 individuals with knee pain at baseline, we found no significant association of weight-bearing or non–weight-bearing activity with incident radiographic knee osteoarthritis for any LMI tertiles (eTable 10 in Supplement 1). Unfortunately, owing to a low number of cases in the low LMI tertile (7 patients), we could not conduct a stratification analysis on LMI for incident symptomatic osteoarthritis. To see whether our results were related to people who already had possible knee degeneration at baseline, we had excluded all participants with a KLG of 1 at baseline and repeated all analyses in population without baseline pain. Compared with the main analysis, this additional analysis showed consistent results: weight-bearing activity was significantly associated with incident radiographic osteoarthritis in the lowest LMI tertile only. Results from models 1 and 2 were statistically significant after multiple testing correction (eTables 11 and 12 in Supplement 1).

## Discussion

This cohort study examined the association of different types of recreational physical activity with incident knee osteoarthritis outcomes and whether this association differs among people with different LMI. We observed associations between higher weight-bearing recreational physical activity and increased odds of incident radiographic knee osteoarthritis in individuals without knee pain. Interestingly, this association seems to be primarily seen in participants with low LMI, not those with middle or high LMI. Also, we did not observe such an association for non–weight-bearing recreational physical activity.

Our results do not contradict the results of the recent meta-analysis^8^ on physical activity, which found no association of total physical activity with knee osteoarthritis. Our study builds on that work, and we identified that only specific activity (weight-bearing) was associated with increased odds of knee osteoarthritis, within a specific subgroup of individuals with low LMI. Moreover, our results align with previous studies on physical activity and osteoarthritis. A previous longitudinal study^24^ of 1678 patients assigned scores to different types of activity on the basis of the mechanical strain they exerted on knee joints. It found that participants doing higher mechanical strain activity were at an increased risk of incident knee osteoarthritis. In addition, a positive association of vigorous activity with knee osteoarthritis risk was identified in the Framingham Study (470 patients).^25^ These findings suggest that activity imposing greater mechanical stress on the knee joint may contribute to the development of knee osteoarthritis. Moreover, a recent systematic review and meta-analysis^13^ encompassing 5707 individuals demonstrated that weakness in knee extensor muscle strength is a risk factor for knee osteoarthritis.

Although we cannot establish a causal relationship on the basis of this observational population study, we hypothesize that the mechanical loading on joints and cartilage could explain the association of weight-bearing activity with osteoarthritis in the low LMI tertile group. During weight-bearing activity, knees are subject to the impact of several times the body weight. For example, the average pressure on knee cartilage is estimated at 10.28 MPa during running.^26^ Such pressure could already constitute a high enough pressure for joint overloading, because this threshold can be quite low. Several studies^27,28,29^ have pointed out that excessive mechanical loading could begin as low as 7 to 9 MPa; 10 MPa is high-impact loading for cartilage that can lead to an increase in matrix metalloproteinases and cause cartilage damage.^29^ Furthermore, biomechanical and cell studies have shown that overloading can trigger several catabolic pathways leading to early osteoarthritis.^27,30^ Therefore, weight-bearing physical activity could harm the knee in certain circumstances, such as low LMI. Yet, muscles and ligaments absorb the kinetic energy generated in activity.^31,32^ By absorbing a part of the impact, the soft tissue reduces the pressure on the cartilage.^12^ A biomechanical study^33^ also showed that better knee muscle activation is associated with less knee cartilage loss after running. Moreover, thigh muscle–specific strength has been suggested to be related to knee osteoarthritis risk.^34^ Yet, the absence of thigh strength data in RS precludes us from assessing whether thigh mass or strength is associated with the risk of knee osteoarthritis. However, a recent cross-sectional study^16^ of 1818 participants did show that knee extension strength is significantly positively correlated with DXA-measured lean mass of the right lower extremity divided by height squared for both men (*r*^2^ = 0.46) and women (*r*^2^ = 0.40; *P* < .001). These studies could explain why no significant association of weight-bearing activity with osteoarthritis was found for individuals in the middle and high LMI tertiles, who have more muscle mass. Taken together, our results, along with the aforementioned findings, suggest that the impact of loading on the joints in people with low LMI might increase knee osteoarthritis odds.

Pain is a complex factor that can greatly mediate the association of physical activity with osteoarthritis. Our current study did not find a statistically significant association of various types of exercise with osteoarthritis in the population with baseline joint pain. However, estimated ORs were similar, suggesting that this analysis was underpowered because of the low sample size. In addition, the inverse causality effect might mediate the association of physical activity with osteoarthritis among those with knee pain.^35^ In our case, inverse causality means that individuals with pain at baseline (ie, those at high osteoarthritis risk) may have reduced their exercise levels because of the pain. This explanation is consistent with our characteristics data, which showed that those with baseline pain had lower physical activity levels than those without pain. Given the intricate nature of these relationships and the inherent limitations of our observational study, future studies with larger sample sizes and different methods, such as randomized clinical trials, are needed to provide more definitive insights.

### Strengths and Limitations

This study has several strengths. First, this study distinguishes between weight-bearing and non–weight-bearing activity, allowing for a more nuanced analysis of the impact of different types of physical activity on knee osteoarthritis. Second, to our knowledge, it is the first large cohort study to examine the role of LMI in the association of physical activity with knee osteoarthritis. Third, the study benefits from DXA data and is embedded within a large, prospective, longitudinal cohort.

This study also has potential limitations. First, RS is a predominantly ethnically nondiverse population cohort, so the result may not be directly generalized to other populations. Second, using questionnaires to collect recreational physical activity data introduces the possibility of recall bias and social desirability bias. These biases could lead to either underestimation or overestimation of recreational physical activity.^36^ Another major limitation of the current study is that knee injury data are unavailable. Knee injury could mediate the results in 2 possible ways. Knee injury could be a confounder between physical activity and osteoarthritis. Participants with knee injuries might have reduced physical activity, and knee injury history itself has been identified as a risk factor for osteoarthritis.^5^ However, if that is the case, we would have found a negative association of physical activity with osteoarthritis, which is inconsistent with current findings. Conversely, knee injury could also act as a mediator between physical activity and osteoarthritis, whereby individuals with higher physical activity levels are more prone to knee injuries, leading to increased odds of osteoarthritis. Furthermore, we only included physical activity at the baseline and lacked data on lifetime physical activity. Like most epidemiology studies, using exposure at baseline leads to the assumption that the participants remain at the same level of exposure during the follow-up period, which might not always be accurate. Lifetime physical activity reflects intensity during working ages and a history of sports participation, an important source of knee injury. Moreover, we were unable to assess whether thigh strength is associated with the risk of knee osteoarthritis because of the absence of directly measured thigh strength data. Furthermore, the subgroup analysis on DXA might be a source of selection bias. In addition, like all longitudinal observational studies, we cannot rule out survivor bias and residual confounding.

## Conclusions

The findings of this cohort study have important clinical implications. Although we did not find an association of recreational physical activity with symptomatic knee osteoarthritis, we did find that weight-bearing activity could contribute to increased odds of radiographic knee osteoarthritis, but only among those with low LMI. Although physical activity is known to have numerous health benefits, our study suggests that caution is needed when engaging in weight-bearing activity, especially for individuals with low levels of lower-limb muscle mass. Although DXA scans are the criterion standard for muscle mass measurement, it might not be feasible to perform DXA scans on all individuals with knee osteoarthritis in the clinic. Lower limb muscle functions can be assessed in other ways such as thigh circumference, which might be a promising avenue for tailored advice for physical activity.

## Notes

Supplement 1. **eAppendix.** Supplemental methods **eTable 1.** Classification of physical activities included **eTable 2.** Physical activities level of the Rotterdam Study sub-cohorts **eTable 3.** Baseline characteristics of population lost in follow-up and population included for analysis **eTable 4.** Baseline characteristics of the study population **eTable 5.** Association of physical activities and incident symptomatic knee osteoarthritis in the pain-free population **eTable 6.** Association between physical activities and incident radiographic knee osteoarthritis in population with baseline knee pain **eTable 7.** Baselines characteristics of population with DXA data and population without **eTable 8.** Baseline characteristics of the subgroup population with available DXA data **eTable 9.** Stratification analysis on tertiles of lower-limb muscle mass index (LMI) for association between physical activities and incident radiographic knee osteoarthritis in population without baseline knee pain **eTable 10.** Stratification analysis on tertiles of lower-limb muscle mass index (LMI) for the association between physical activities and incident radiographic knee osteoarthritis in population with baseline knee pain **eTable 11.** Association between physical activities and incident radiographic knee osteoarthritis in populations without baseline knee pain and excluding baseline KLG>=1 **eTable 12.** Association between physical activities and incident radiographic knee osteoarthritis in populations excluding baseline KLG>=1
eReferences

Supplement 2.
Data Sharing Statement

## References

[1] Prieto-Alhambra D, Judge A, Javaid MK, Cooper C, Diez-Perez A, Arden NK. Incidence and risk factors for clinically diagnosed knee, hip and hand osteoarthritis: influences of age, gender and osteoarthritis affecting other joints. Ann Rheum Dis. 2014;73(9):1659-1664. doi:10.1136/annrheumdis-2013-203355 23744977 PMC3875433

[2] Hunter DJ, Schofield D, Callander E. The individual and socioeconomic impact of osteoarthritis. Nat Rev Rheumatol. 2014;10(7):437-441. doi:10.1038/nrrheum.2014.44 24662640

[3] Barbour KE, Helmick CG, Theis KA, ; Centers for Disease Control and Prevention (CDC). Prevalence of doctor-diagnosed arthritis and arthritis-attributable activity limitation–United States, 2010-2012. MMWR Morb Mortal Wkly Rep. 2013;62(44):869-873. 24196662 PMC4585589

[4] Felson DT, Zhang Y, Hannan MT, . Risk factors for incident radiographic knee osteoarthritis in the elderly: the Framingham Study. Arthritis Rheum. 1997;40(4):728-733. doi:10.1002/art.1780400420 9125257

[5] Hunter DJ, Bierma-Zeinstra S. Osteoarthritis. Lancet. 2019;393(10182):1745-1759. doi:10.1016/S0140-6736(19)30417-9 31034380

[6] Lin W, Alizai H, Joseph GB, . Physical activity in relation to knee cartilage T2 progression measured with 3 T MRI over a period of 4 years: data from the Osteoarthritis Initiative. Osteoarthritis Cartilage. 2013;21(10):1558-1566. doi:10.1016/j.joca.2013.06.022 23831632 PMC3874212

[7] Doré DA, Winzenberg TM, Ding C, . The association between objectively measured physical activity and knee structural change using MRI. Ann Rheum Dis. 2013;72(7):1170-1175. doi:10.1136/annrheumdis-2012-201691 22896739

[8] Gates LS, Perry TA, Golightly YM, . Recreational physical activity and risk of incident knee osteoarthritis: an international meta-analysis of individual participant-level data. Arthritis Rheumatol. 2022;74(4):612-622. doi:10.1002/art.42001 34730279 PMC9450021

[9] Verweij LM, van Schoor NM, Dekker J, Visser M. Distinguishing four components underlying physical activity: a new approach to using physical activity questionnaire data in old age. BMC Geriatr. 2010;10:20. doi:10.1186/1471-2318-10-20 20438623 PMC2873369

[10] Grawe B, Schroeder AJ, Kakazu R, Messer MS. Lateral collateral ligament injury about the knee: anatomy, evaluation, and management. J Am Acad Orthop Surg. 2018;26(6):e120-e127. doi:10.5435/JAAOS-D-16-00028 29443704

[11] Flandry F, Hommel G. Normal anatomy and biomechanics of the knee. Sports Med Arthrosc Rev. 2011;19(2):82-92. doi:10.1097/JSA.0b013e318210c0aa 21540705

[12] van Veen B, Montefiori E, Modenese L, Mazzà C, Viceconti M. Muscle recruitment strategies can reduce joint loading during level walking. J Biomech. 2019;97:109368. doi:10.1016/j.jbiomech.2019.109368 31606129

[13] Øiestad BE, Juhl CB, Culvenor AG, Berg B, Thorlund JB. Knee extensor muscle weakness is a risk factor for the development of knee osteoarthritis: an updated systematic review and meta-analysis including 46 819 men and women. Br J Sports Med. 2022;56(6):349-355. doi:10.1136/bjsports-2021-104861 34916210

[14] Patterson BE, Girdwood MA, West TJ, . Muscle strength and osteoarthritis of the knee: a systematic review and meta-analysis of longitudinal studies. Skeletal Radiol. 2023;52(11):2085-2097. doi:10.1007/s00256-022-04266-4 36562820

[15] Ikram MA, Kieboom BCT, Brouwer WP, . The Rotterdam Study: design update and major findings between 2020 and 2024. Eur J Epidemiol. 2024;39(2):183-206. doi:10.1007/s10654-023-01094-1 38324224

[16] Tsukasaki K, Matsui Y, Arai H, . Association of muscle strength and gait speed with cross-sectional muscle area determined by mid-thigh computed tomography: a comparison with skeletal muscle mass measured by dual-energy x-ray absorptiometry. J Frailty Aging. 2020;9(2):82-89. doi:10.14283/jfa.2020.1632259181

[17] Stel VS, Smit JH, Pluijm SM, Visser M, Deeg DJ, Lips P. Comparison of the LASA Physical Activity Questionnaire with a 7-day diary and pedometer. J Clin Epidemiol. 2004;57(3):252-258. doi:10.1016/j.jclinepi.2003.07.008 15066685

[18] Caspersen CJ, Bloemberg BPM, Saris WHM, Merritt RK, Kromhout D. The prevalence of selected physical activities and their relation with coronary heart disease risk factors in elderly men: the Zutphen Study, 1985. Am J Epidemiol. 1991;133(11):1078-1092. doi:10.1093/oxfordjournals.aje.a115821 2035512

[19] Koolhaas CM, Dhana K, Schoufour JD, . Physical activity and cause-specific mortality: the Rotterdam Study. Int J Epidemiol. 2018;47(5):1705-1713. doi:10.1093/ije/dyy058 29672692

[20] Borga M, West J, Bell JD, . Advanced body composition assessment: from body mass index to body composition profiling. J Investig Med. 2018;66(5):1-9. doi:10.1136/jim-2018-000722 29581385 PMC5992366

[21] Shepherd JA, Ng BK, Sommer MJ, Heymsfield SB. Body composition by DXA. Bone. 2017;104:101-105. doi:10.1016/j.bone.2017.06.010 28625918 PMC5659281

[22] Benjamini Y, Hochberg Y. Controlling the false discovery rate: a practical and powerful approach to multiple testing. J R Stat Soc B. 1995;57(1):289-300. doi:10.1111/j.2517-6161.1995.tb02031.x

[23] Holden MA, Nicholls EE, Young J, Hay EM, Foster NE. Exercise and physical activity in older adults with knee pain: a mixed methods study. Rheumatology (Oxford). 2015;54(3):413-423. doi:10.1093/rheumatology/keu333 25187640 PMC4334683

[24] Verweij LM, van Schoor NM, Deeg DJ, Dekker J, Visser M. Physical activity and incident clinical knee osteoarthritis in older adults. Arthritis Rheum. 2009;61(2):152-157. doi:10.1002/art.24233 19177523

[25] McAlindon TE, Wilson PW, Aliabadi P, Weissman B, Felson DT. Level of physical activity and the risk of radiographic and symptomatic knee osteoarthritis in the elderly: the Framingham study. Am J Med. 1999;106(2):151-157. doi:10.1016/S0002-9343(98)00413-6 10230743

[26] Sinclair J. Effects of barefoot and barefoot inspired footwear on knee and ankle loading during running. Clin Biomech (Bristol, Avon). 2014;29(4):395-399. doi:10.1016/j.clinbiomech.2014.02.004 24636307

[27] Hosseini SM, Veldink MB, Ito K, van Donkelaar CC. Is collagen fiber damage the cause of early softening in articular cartilage? Osteoarthritis Cartilage. 2013;21(1):136-143. doi:10.1016/j.joca.2012.09.002 23010079

[28] Mononen ME, Tanska P, Isaksson H, Korhonen RK. A novel method to simulate the progression of collagen degeneration of cartilage in the knee: data from the Osteoarthritis Initiative. Sci Rep. 2016;6:21415. doi:10.1038/srep21415 26906749 PMC4764929

[29] Leong DJ, Li YH, Gu XI, . Physiological loading of joints prevents cartilage degradation through CITED2. FASEB J. 2011;25(1):182-191. doi:10.1096/fj.10-164277 20826544 PMC3005439

[30] Jørgensen AEM, Kjær M, Heinemeier KM. The effect of aging and mechanical loading on the metabolism of articular cartilage. J Rheumatol. 2017;44(4):410-417. doi:10.3899/jrheum.160226 28250141

[31] Coobs BR, LaPrade RF, Griffith CJ, Nelson BJ. Biomechanical analysis of an isolated fibular (lateral) collateral ligament reconstruction using an autogenous semitendinosus graft. Am J Sports Med. 2007;35(9):1521-1527. doi:10.1177/0363546507302217 17495013

[32] Buzzi R, Aglietti P, Vena LM, Giron F. Lateral collateral ligament reconstruction using a semitendinosus graft. Knee Surg Sports Traumatol Arthrosc. 2004;12(1):36-42. doi:10.1007/s00167-003-0456-6 14615886

[33] Kersting UG, Stubendorff JJ, Schmidt MC, Brüggemann GP. Changes in knee cartilage volume and serum COMP concentration after running exercise. Osteoarthritis Cartilage. 2005;13(10):925-934. doi:10.1016/j.joca.2005.06.005 16154364

[34] Culvenor AG, Felson DT, Niu J, . Thigh muscle specific-strength and the risk of incident knee osteoarthritis: the influence of sex and greater body mass index. Arthritis Care Res (Hoboken). 2017;69(8):1266-1270. doi:10.1002/acr.23182 28176489 PMC5532059

[35] Morgan SL, Winship C. Counterfactuals and Causal Inference. Cambridge University Press; 2015.

[36] Sallis JF, Saelens BE. Assessment of physical activity by self-report: status, limitations, and future directions. Res Q Exerc Sport. 2000;71(suppl 2):1-14. doi:10.1080/02701367.2000.11082780 25680007

