# Supplementary material for: Weight-Bearing Physical Activity, Lower-Limb Muscle Mass, and Risk of Knee Osteoarthritis
Source: JAMA Netw Open. 2024 Apr 30;7(4):e248968. doi: 10.1001/jamanetworkopen.2024.8968 (PMC11061770; doi:10.1001/jamanetworkopen.2024.8968)
Supplement: Supplement 1. — eAppendix. Supplemental methods eTable 1. Classification of physical activities included eTable 2. Physical activities level of the Rotterdam Study sub-cohorts eTable 3. Baseline characteristics of population lost in follow-up and population included for analysis eTable 4. Baseline characteristics of the study population eTable 5. Association of physical activities and incident symptomatic knee osteoarthritis in the pain-free population eTable 6. Association between physical activities and incident radiographic knee osteoarthritis in population with baseline knee pain eTable 7. Baselines characteristics of population with DXA data and population without eTable 8. Baseline characteristics of the subgroup population with available DXA data eTable 9. Stratification analysis on tertiles of lower-limb muscle mass index (LMI) for association between physical activities and incident radiographic knee osteoarthritis in population without baseline knee pain eTable 10. Stratification analysis on tertiles of lower-limb muscle mass index (LMI) for the association between physical activities and incident radiographic knee osteoarthritis in population with baseline knee pain eTable 11. Association between physical activities and incident radiographic knee osteoarthritis in populations without baseline knee pain and excluding baseline KLG>=1 eTable 12. Association between physical activities and incident radiographic knee osteoarthritis in populations excluding baseline KLG>=1 eReferences [file jamanetwopen-e248968-s001.pdf]

Supplemental Online Content

Wu Y, Boer CG, Hofman A, et al. Weight-bearing physical activity, lower-limb muscle mass, and risk of knee osteoarthritis. *JAMA Netw Open*. 2024;7(4):e248968. doi:10.1001/jamanetworkopen.2024.8968

eAppendix. Supplemental Methods

eTable 1. Classification of physical activities included

eTable 2. Physical activities level of the Rotterdam Study sub-cohorts

eTable 3. Baseline characteristics of population lost in follow-up and population included for analysis

eTable 4. Baseline characteristics of the study population

eTable 5. Association of physical activities and incident symptomatic knee osteoarthritis in the pain-free population

eTable 6. Association between physical activities and incident radiographic knee osteoarthritis in population with baseline knee pain

eTable 7. Baselines characteristics of population with for DXA data and population without

eTable 8. Baseline characteristics of the subgroup population with available DXA data

eTable 9. Stratification analysis on tertiles of lower-limb muscle mass index (LMI) for association between physical activities and incident radiographic knee osteoarthritis in population without baseline knee pain

eTable 10. Stratification analysis on tertiles of lower-limb muscle mass index (LMI) for the association between physical activities and incident radiographic knee osteoarthritis in population with baseline knee pain

eTable 11. Association between physical activities and incident radiographic knee osteoarthritis in populations without baseline knee pain and excluding baseline KLG>=1

eTable 12. Association between physical activities and incident radiographic knee osteoarthritis in populations excluding baseline KLG>=1

eReferences

This supplemental material has been provided by the authors to give readers additional information about their work.

© 2024 Wu Y et al. *JAMA Netw Open.*

eAppendix. Supplemental Methods

Study design and population

This study was embedded in the Rotterdam Study (RS), a large population-based prospective cohort study started in 1990. The design of the Rotterdam Study has been previously described in detail[1] (Figure 1). The Rotterdam Study has been approved by the Medical Ethics Committee of the Erasmus MC (registration number MEC 02.1015) and by the Dutch Ministry of Health, Welfare and Sport (Population Screening Act WBO, license number 1071272-159521-PG). As a longitudinal population-based cohort Study, the Rotterdam Study has no pre-specified health exclusion criteria, meaning that all persons older than 55 years of age living in the area were invited to participate. The current analysis included 5003 participants from the three RS sub-cohorts (RS-I, RS-II, and RS-III) who had complete data of baseline recreational physical activity, baseline knee pain, and knee radiographs from baseline and at least one time from follow-up visits Participants with x-ray-defined osteoarthritis for one or both knees at baseline were excluded (Figure 2).

The observation periods are 1997 to 2002 for RS-I, 2000 to 2011 for RS-II, and 2006 to 2012 for RS-III, with an average of 6.3 years follow-up after baseline. Participants of RS-I and RS-II underwent two follow-up evaluations, while those in RS-III while those in RS-III had one follow-up assessment post-baseline.

Recreational physical activity

We assessed physical activity as an exposure variable using two validated questionnaires: an adapted version of the Zutphen Physical Activity Questionnaire for RS-I and RS-II and the Longitudinal Aging Study Amsterdam (LASA) physical activity questionnaire for RS-III[2,3]. Both questionnaires asked participants about the frequency and duration of various types of physical activity. For RS-I and RS-II participants, physical activity information was gathered using the Zutphen questionnaire, covering the two weeks preceding the interview. In contrast, participants in RS-III, interviewed with the LASA questionnaire, had their physical activity data collected separately for winter and summer periods and expressed as averages. The metabolic equivalent of task (MET)[4], defined as the ratio of the rate of energy expended during an activity to the rate of energy expended at rest, was used to weigh the intensity of physical activity and all activities were expressed in MET*hours/week.

Based on data from a previous biomechanical study, physical activity levels were divided and summarized into weight-bearing and non-weight-bearing physical activity levels [5]. Non-weight-bearing activities were defined as those in which the knee joint did not bear the body weight, while weight-bearing activities were defined as those in which the knee joint did bear the body weight. All activities in the questionnaires were classified into weight-bearing or non-weight-bearing activities, except for household work. Because household work is an umbrella term that covers multiple activities, both weight-bearing and non-weight-bearing, we could not distinguish. The detailed classification of physical activities is listed in Supplementary eTable 1. Total physical activity, weight-bearing physical activity and non-weight-bearing physical activity, all expressed in MET*hours/week, of each participant were the three exposures of this study. Total physical activity was defined as the combination of weight-bearing and non-weight-bearing activities. Since physical activity was assessed using different questionnaires across the RS cohorts. total physical activity, weight-bearing physical activity and non-weight-bearing physical activity were standardized into Z-scores per cohort. The original MET*hours/week physical activity data each unit represents after standardization are summarized in supplementary eTable 2.

Co-variates

We have used the following co-variates: Age, sex, BMI, baseline KLG, RS sub-cohorts, education level, alcohol intake, smoking, systolic blood pressure, HDL/total cholesterol ratio, and diabetes mellitus prevalence. Co-variates data were collected at baseline: age (years), education level (Primary education, Lower/intermediate general or lower vocational education, Intermediate vocational or higher general education, and Higher vocational education or university), alcohol intake (g/day), and smoking (Current smoker. former smoker, never smoker) were measured in-home interviews. BMI (kg/m^2), systolic blood pressure (mmHg), and HDL/total cholesterol ratio were measured at the research center. Diabetes mellitus prevalence data were collected by consulting medical records. The missing rate of all co-variates was below 3.4% except for alcohol intake (8.6%), and missing values were imputed by using the multiple imputation function from the MICE package in R[6]. Parameter settings (datasets=10, iteration=5)[7].

Osteoarthritis

We assessed two outcomes: incident radiographic knee osteoarthritis based on x-ray radiographs and incident symptomatic knee osteoarthritis based on a self-reported pain questionnaire and x-ray radiographs. Radiographic knee osteoarthritis is the primary outcome and symptomatic knee osteoarthritis is secondary outcome.

Incident radiographic knee osteoarthritis

© 2024 Wu Y et al. *JAMA Netw Open.*

Weight-bearing anteroposterior radiographs of the knee and hip were obtained at 70 kV, a focus of 1.8, and a focus to film distance of 120 cm, applying a Fuji high-resolution G 35×43 cm film. The knee was fully extended with the patella in a central position. Incident radiographic knee osteoarthritis is assessed using the Kellgren and Lawrence Grade (KLG) in knee x-ray radiographs[8,9]. The radiographs at baseline and follow-up were read without knowledge of the participants' clinical status, the research hypothesis, or the exposure status of the participants. Left and right knee radiographs were grouped per subject and read in pairs. The x-rays of the same subject from the baselines and follow-up were not paired for x-ray readers. The radiographs were scored by a group of trained readers, the interrater reliability was 0.71. We defined incident radiographic knee osteoarthritis as one knee having baseline KLG<2 and follow-up KLG≥2 or having a knee replacement at follow-up. Incident knee osteoarthritis was determined separately for each participant's left and right knee.

Incident symptomatic knee osteoarthritis

Knee pain data was collected through questionnaire-based interviews, where participants were asked if they had experienced pain in their knees in the past month. Incident symptomatic knee osteoarthritis is defined as a participant having no knee pain and no knee osteoarthritis (KLG<2) in both knees (left and right) at baseline, and in follow-up had radiographic osteoarthritis (KLG≥2) or joint replacement in least one knee at follow up and reported knee pain in the questionnaire.

Measurements of lower-limb muscle mass

Lower-limb muscle mass was measured by dual X-ray Absorptiometry (Prodigy and iDXA devices, GE Healthcare, Chicago, United States)[11,12], in a randomly selected group (n=1881) of sub-cohorts of RS (RS-II and RS-III). To adjust for height, we calculate a lower-limb muscle mass index (LMI) as lower limb lean mass in kilograms (*kg*) divided by height(*m*) in meters squared (kg/m^2). This index method aligns with previous literature [13]. We standardized the index using sex-specific Z-scores to consider the difference in LMI between men and women.

Statistical analysis

The relationship between recreational physical activity at baseline (total, weight-bearing, and non-weight-bearing) and incidence of radiographic osteoarthritis and symptomatic osteoarthritis was assessed using logistic regression, adjusted for RS sub-cohorts, baseline KLG, follow-up time, age, sex, and BMI (model 1). When analyzing incident radiographic knee osteoarthritis, both knees from the same individual were included. A generalized estimating equation (GEE) was used to account for the correlation of the knees from the same participant. We also adjusted for education level, alcohol intake, smoking, systolic blood pressure, HDL/total cholesterol ratio, and diabetes mellitus prevalence (model 2). To study the influence of LMI on the relation between recreational physical activity and osteoarthritis, a pre-specified stratification analysis based on tertiles of LMI was conducted in the subgroup of participants with LMI data available (n=1881). To reduce the chance of type I error, Benjamin-Hochberg multiple testing correction was applied to all analysis results with false discovery rate of 0.05[10]. All analyses were done separately in participants with baseline pain and without. All statistical analyses were performed using R (version 4.2.1, R Foundation for Statistical Computing, Vienna, Austria).

Equity, diversity and inclusion statement

The authors include women and men in biology and clinical specialties from Europe and Asia. The study population included a spectrum of demographics. In discussing the generalizability and limitations of the findings, we acknowledge that RS is an ethnically non-diverse population cohort.

© 2024 Wu Y et al. *JAMA Netw Open.*

eTable 1. Classification of physical activities included

Non-weight bearing activities	Weight-bearing activities
Cycling,	Walking
Rowing,	Gardening
Swimming	Golf
Cycling sport,	Jogging
Fishing	Sailing
	Dancing
	Ball sports
	Tennis
	Ice skating
	Moving to music
	Gymnastics
	Bowling
	Winter sports

Physical activity is assessed by the Zutphen and LASA questionnaire. Classification is based on a previous biomechanical study in the LASA questionnaire[5].

© 2024 Wu Y et al. *JAMA Netw Open.*

eTable 2. Physical activities level of the Rotterdam Study sub-cohorts

	**Total physical activities, MET*Hour/week, mean (SD)**	**Weight-bearing activities, MET*Hour/week, mean (SD)**	**Non-Weight-bearing activities, MET*Hour/week, mean (SD)**
**Rotterdam study sub-cohort 1**	43.46 (35.83)	35.41(31.19)	11.38(14.92)
**Rotterdam study sub-cohort 2**	40.92(29.25)	29.55(22.01)	8.05(13.57)
**Rotterdam study sub-cohort 3**	22.52(37.66)	15.69(31.13)	6.83(15.64)

*Data are presented in mean and Standard deviations. Unit is MET*Hour per week. MET= Metabolic equivalent of task.*

© 2024 Wu Y et al. *JAMA Netw Open.*

eTable 3. Baseline characteristics of population lost in follow-up and population included for analysis

	No (%)	
	Population lost in follow up	Population included for analysis
N	2224	5003
**sex (n, %)**		
Men	1032 (46.4)	2199 (44.0)
Women	1192 (53.6)	2804 (56.0)
**Age, mean (SD), years**	67.35(11.24)	64.48(7.89)
**Rotterdam study sub-cohorts (n, %)**		
RS-I	994 (44.7)	2172 (43.4)
RS-II	444 (20.0)	1470 (29.4)
RS-III	786 (35.3)	1361 (27.2)
**Follow-up Time, mean (SD), years**	5.90 (1.73)	6.33 (2.46)
**BMI, mean (SD), kg/m^2**	26.82 (4.12)	26.95 (3.92)
**Lower limb muscle index, mean (SD), kg/m^2**	5.60 (0.83)	5.57 (0.85)
**Education level (n, %)**		
Primary education	318 (14.4)	502 (10.1)
Lower/intermediate general or lower vocational education	876 (39.6)	2076 (41.8)
Intermediate vocational or higher general education	618 (28.0)	1483 (29.8)
Higher vocational education or university	398 (18.0)	908 (18.3)
**Smoking (n, %)**		
Never smoker	816 (36.7)	2064 (41.3)
Former smoker	1064 (47.8)	2335 (46.7)
Current smoker	344 (15.5)	603 (12.1)
**Alcohol intake, mean (SD), g/day**	10.42 (14.06)	11.19 (14.16)
**HDL/total cholesterol ratio, mean (SD)**	0.25 (0.08)	0.25 (0.08)
**Systolic blood pressure, mean (SD), mmHg**	141.71 (22.59)	139 (21)
**Diabetes at baseline (n, %)**	319 (14.3)	497 (9.9)
**Baseline KLG=1 (n, %)**	832(37.4)	1587 (31.7)
**Total physical activity, mean (SD), MET*Hour/week**	36.88 (32.93)	43.6 (33.6)
**Weight-bearing physical activity, mean (SD), MET*Hour/week**	28.63 (27.75)	32.4 (27.7)
**Non-weight-bearing physical activity, mean (SD), MET*Hour/week**	8.25 (14.26)	11.2 (15.8)

*BMI= Body mass index, HDL= high-density lipoprotein. RS= Rotterdam study. KLG= the Kellgren and Lawrence Grade (KLG). MET*Hour/week = Hours of Metabolic equivalent of task per week. Continuous data are shown in mean with Standard deviations, and category variables are shown in the number of cases and percentage.*

© 2024 Wu Y et al. *JAMA Netw Open.*

eTable 4. Baseline characteristics of the study population

	No (%)						
	Total study populatio	Study Population without baseline knee pai			Population with baseline pain		
		Total	Male	Female	Total	Male	Female
**N**	5003	3492	1684	1808	1511	515	996
**Sex (n, %)**	2804 (56.0)						
**Men**	2199 (44.0)						
**Women**	2804 (56.0)						
**Age, mean (SD), years**	64.48(7.89)	64.75 (7.97)	64.88 (7.83)	64.63 (8.11)	63.86 (7.67)	64.14 (7.43)	63.71 (7.78)
**Rotterdam study sub-cohorts (n, %)**							
**RS-I**	2172 (43.4)	1642 (47.0)	812 (48.2)	830 (45.9)	530 (35.1)	180 (35.0)	350 (35.1)
**RS-II**	1470 (29.4)	912 (26.1)	457 (27.1)	455 (25.2)	558 (36.9)	203 (39.4)	355 (35.6)
**RS-III**	1361 (27.2)	938 (26.9)	415 (24.6)	523 (28.9)	423 (28.0)	132 (25.6)	291 (29.2)
**Follow-up Time, mean (SD), years**	6.33 (2.46)	6.15 (2.38)	6.19 (2.44)	6.12 (2.32)	6.73 (2.61)	6.81 (2.70)	6.69 (2.56)
**BMI, mean (SD), kg/m^2**	26.95 (3.92)	26.65 (3.72)	26.64 (3.28)	26.65 (4.09)	27.64 (4.27)	27.59 (3.50)	27.67 (4.61)
**Lower limb muscle index, mean (SD), kg/m^2**	5.57 (0.85)	5.60 (0.86)	6.30 (0.61)	5.03 (0.56)	5.52 (0.81)	6.36 (0.64)	5.10 (0.51)
**Education level (n, %)**							
**Primary education**	502 (10.1)	326 (9.4)	116 (6.9)	210 (11.7)	176 (11.7)	45 (8.8)	131 (13.3)
**Lower/intermediate general or lower vocational education**	2076 (41.8)	1419 (40.9)	475 (28.4)	944 (52.6)	657 (43.8)	143 (27.9)	514 (52.0)
**Intermediate vocational or higher general education**	1483 (29.8)	1064 (30.7)	625 (37.3)	439 (24.5)	419 (27.9)	203 (39.6)	216 (21.9)
**Higher vocational education or university**	908 (18.3)	660 (19.0)	459 (27.4)	201 (11.2)	248 (16.5)	121 (23.6)	127 (12.9)
**Smoking (n, %)**							
**Never smoker**	2064 (41.3)	1360 (39.0)	434 (25.8)	926 (51.2)	704 (46.6)	153 (29.7)	551 (55.3)
**Former smoker**	2335 (46.7)	1673 (47.9)	1043 (61.9)	630 (34.9)	662 (43.8)	312 (60.6)	350 (35.1)
**Current smoker**	603 (12.1)	458 (13.1)	207 (12.3)	251 (13.9)	145 (9.6)	50 (9.7)	95 (9.5)
**Alcohol intake, mean (SD), g/day**	11.19 (14.16)	11.25 (13.49)	15.03 (15.52)	7.73 (10.06)	11.03 (15.65)	17.62 (21.22)	7.70 (10.40)
**HDL/total cholesterol ratio, mean (SD)**	0.25 (0.08)	0.25 (0.08)	0.23 (0.07)	0.27 (0.08)	0.25 (0.08)	0.23 (0.07)	0.26 (0.08)
**Systolic blood pressure, mean (SD), mmHg**	139 (21)	140 (21)	141(20)	138.(21)	138 (20)	140(19)	137 (21)
**Diabetes at baseline (n, %)**	497 (9.9)	351 (10.1)	204 (12.1)	147 (8.1)	146 (9.7)	66 (12.8)	80 (8.0)
**Baseline KLG=1 (n, %)**	1587 (31.7)	1024 (29.3)	460 (27.3)	564 (31.2)	563 (37.3)	179 (34.8)	384 (38.6)

© 2024 Wu Y et al. *JAMA Netw Open.*

**Total physical activity, mean (SD), MET*Hour/week**	43.6 (33.6)	44.5 (34.5)	46.7 (36.2)	42.5 (32.8)	41.6 (31.1)	43.2 (33.7)	40.7 (29.6)
**Weight-bearing physical activity, mean (SD), % of total physical activity**	76% (25%)	77% (24%)	76% (24%)	78% (24%)	74% (26%)	72% (26%)	75% (26%)
**Non-weight-bearing physical activity, mean (SD), % of total physical activity**	24% (25%)	23% (24%)	24% (24%)	22% (24%)	26% (26%)	28% (26%)	25% (26%)
**Weight-bearing physical activity, mean (SD), MET*Hour/week**	32.4 (27.7)	33.5 (28.7)	34.2 (29.2)	32.8 (28.3)	29.94(24.9)	29.5 (25.0)	30.2 (24.9)
**Sports activity (i.e. Running), mean (SD), % of Weight-bearing physical activity**	11% (23)	11% (23%)	12% (24%)	10% (22%)	10% (22)	9% (22%)	10% (22%)
**Daily activity (i.e. Walking), mean (SD), % of weight-bearing physical activity**	89% (23%)	89% (23%)	88% (24%)	90% (22%)	90% (22%)	91% (22%)	90% (22%)
**Non-weight-bearing physical activity, mean (SD), MET*Hour/week**	11.2 (15.8)	11.0 (15.7)	12.4 (17.2)	9.71 (13.9 9)	11.6 (16.2)	13.7 (19.4)	10.5 (14.2)
**Sports activity (i.e. Swimming), mean (SD), % of non-weight-bearing physical activity**	14% (29%)	14% (29%)	13% (29%)	14% (29%)	13% (28%)	13% (28%)	13% (28%)
**Daily activity (i.e. Biking) , mean (SD), % of non-weight-bearing physical activity**	86% (29%)	86% (29%)	87% (29%)	86% (29%)	87% (28%)	87% (28%)	87% (28%)

*BMI= Body mass index, HDL= high-density lipoprotein. RS= Rotterdam study. KLG= the Kellgren and Lawrence Grade (KLG). MET*Hour/week = Hours of Metabolic equivalent of task per week. Continuous data are shown in mean with Standard deviations, and category variables are shown in the number of cases and percentage.*

© 2024 Wu Y et al. *JAMA Netw Open.*

eTable 5: Association of physical activities and incident symptomatic knee osteoarthritis in the pain-free population

Outcomes	Exposure	Unadjusted model		Model 1		Model 2	
		**OR 95%CI**	**P value**	**OR 95%CI**	**P value**	**OR 95%CI**	**P value**
N = 3473, N with Incident symptomatic osteoartritis (%) = 126 (3.62%)	Total physical activities	1.02(0.85-1.22)	0.82	1.09(0.91-1.31)	0.344	1.09(0.91-1.31)	0.35
	Non-Weight-bearing activities	0.94(0.79-1.13)	0.53	1.00(0.83-1.19)	0.98	1.00(0.83-1.19)	0.97
	Weight-bearing activities	1.06(0.89-1.26)	0.51	1.12(0.94-1.33)	0.22	1.12(0.93-1.33)	0.233

*The statistical model used is a multivariate logistic regression model—Model1 adjusted for age, sex, Rotterdam study sub-cohorts, BMI, follow up time, and baseline KLG. Model 2 additionally adjusted for education level, alcohol intake, smoking, systolic blood pressure, HDL/total cholesterol ratio, and diabetes mellitus. OR= Odds ratio, CI= confidence interval. * Indicates p-value remain significant after multiple testing corrections using Benjamini and Hochberg method.*

© 2024 Wu Y et al. *JAMA Netw Open.*

eTable 6. Association between physical activities and incident radiographic knee osteoarthritis in population with baseline knee pain

Outcomes	Exposure	Unadjusted model		Model 1		Model 2	
		OR 95%CI	P value	OR 95%CI	P value	OR 95%CI	P value
N = 1511, N knee Joints = 2758, N joints with radiographic osteoarthritis incidence (%) = 378 (13.71%)	Total physical activities	1.13(0.98-1.29)	0.08	1.14(0.99-1.32)	0.07	1.15(0.99-1.33)	0.06
	Non-Weight-bearing activities	1.05(0.94-1.17)	0.42	1.08(0.96-1.21)	0.21	1.08(0.96-1.22)	0.22
	Weight-bearing activities	1.12(0.98-1.28)	0.10	1.11(0.96-1.29)	0.17	1.12(0.96-1.3)	0.15

The statistical model used is a multivariate logistic regression model—Model1 adjusted for age, sex, Rotterdam study sub-cohorts, BMI, follow up time, and baseline KLG. Model 2 additionally adjusted for education level, alcohol intake, smoking, systolic blood pressure, HDL/total cholesterol ratio, and diabetes mellitus. OR= Odds ratio, CI= confidence interval. * *Indicates p-value remain significant after multiple testing corrections using Benjamini and Hochberg method.*

© 2024 Wu Y et al. *JAMA Netw Open.*

eTable 7. Baselines characteristics of population with for DXA data and population without

	No (%)	
	**population without DXA data**	**population with DXA data**
**N**	3122	1881
**Sex (n, %)**		
**Men**	1432 (45.9)	767 (40.8)
**Women**	1690 (54.1)	1114 (59.2)
**Age, mean (SD), years**	67.69 (7.18)	59.15 (5.87)
**Rotterdam study sub-cohorts (n, %)**		
**RS-I**	2172 (69.6)	0 (0.0)
**RS-II**	778 (24.9)	692 (36.8)
**RS-III**	172 (5.5)	1189 (63.2)
**Follow-up Time, mean (SD), years**	8.73 (2.57)	7.13 (2.34)
**BMI, mean (SD), kg/m^2**	26.93 (3.93)	26.98 (3.92)
**Education level (n, %)**		
**Primary education**	355 (11.5)	147 (7.9)
**Lower/intermediate general or lower vocational education**	1307 (42.2)	769 (41.1)
**Intermediate vocational or higher general education**	972 (31.4)	511 (27.3)
**Higher vocational education or university**	465 (15.0)	443 (23.7)
**Smoking (n, %)**		
**Never smoker**	1209 (38.7)	855 (45.5)
**Former smoker**	1532 (49.1)	803 (42.7)
**Current smoker**	381 (12.2)	222 (11.8)
**Alcohol intake, mean (SD), g/day**	11.55 (15.38)	10.57 (11.76)
**HDL/total cholesterol ratio, mean (SD)**	0.24 (0.07)	0.26 (0.08)
**Systolic blood pressure, mean (SD), mmHg**	141.60 (20.85)	134.82 (19.49)
**Diabetes at baselines (n, %)**	342 (11.0)	155 (8.2)
**Baselines KLG=1 (n, %)**	1139 (36.5)	448 (23.8)
**Total physical activity, mean (SD), MET*Hour/week**	47.70 (34.46)	36.86 (30.84)
**Weight-bearing physical activity, mean (SD), MET*Hour/week**	36.38 (29.22)	25.85 (23.53)
**Non-weight-bearing physical activity, mean (SD), MET*Hour/week**	11.31 (15.20)	11.01 (16.83)

*DXA= dual X-ray Absorptiometry, BMI= Body mass index, HDL= high-density lipoprotein. RS= Rotterdam study. KLG= the Kellgren and Lawrence Grade (KLG). MET*Hour/week = Hours of Metabolic equivalent of task per week. Continuous data are shown in mean with Standard deviations, and category variables are shown in the number of cases and percentage.*

© 2024 Wu Y et al. *JAMA Netw Open.*

eTable 8: Baseline characteristics of the subgroup population with available DXA data

	No (%)		
	**Total study population**	**Study population without baseline knee pain**	**Study population with baseline knee pain**
**N**	1881	1273	608
**Sex (n, %)**			
**Men**	767 (40.8)	565 (44.4)	202 (33.2)
**Women**	1114 (59.2)	708 (55.6)	406 (66.8)
**Age, mean (SD), years**	59.20 (5.87)	59.07 (6.00)	59.31 (5.58)
**Rotterdam study sub-cohorts (n, %)**			
**RS-II**	692 (36.8)	446 (35.0)	246 (40.5)
**RS-III**	1189 (63.2)	827 (65.0)	362 (59.5)
**Follow-up Time, mean (SD), years**	7.13 (2.34)	7.05 (2.31)	7.29 (2.39)
**BMI, mean (SD), kg/m^2**	26.98 (3.92)	26.70 (3.75)	27.56 (4.19)
**Lower limb muscle index, mean (SD), kg/m^2**	5.57 (0.85)	5.60 (0.86)	5.52 (0.81)
**Education level (n, %)**			
**Primary education**	147 (7.9)	89 (7.0)	58 (9.6)
**Lower/intermediate general or lower vocational education**	769 (41.1)	510 (40.3)	259 (42.8)
**Intermediate vocational or higher general education**	511 (27.3)	351 (27.7)	160 (26.4)
**Higher vocational education or university**	443 (23.7)	315 (24.9)	128 (21.2)
**Smoking (n, %)**			
**Never smoker**	855 (45.5)	564 (44.3)	291 (47.9)
**Former smoker**	803 (42.7)	556 (43.7)	247 (40.6)
**Current smoker**	222 (11.8)	152 (11.9)	70 (11.5)
**Alcohol intake, mean (SD), g/day**	10.57 (11.76)	10.54 (11.52)	10.61 (12.27)
**HDL/total cholesterol ratio, mean (SD)**	0.26 (0.08)	0.26 (0.08)	0.26 (0.08)
**Systolic blood pressure, mean (SD), mmHg**	135 (19)	135 (20)	134 (19)
**Diabetes at baselines (n, %)**	155 (8.2)	106 (8.3)	49 (8.1)
**Baselines KLG=1 (n, %)**	448 (23.8)	260 (20.4)	188 (30.9)
**Total physical activity, mean (SD), MET*Hour/week**	36.86 (30.84)	37.03 (32.24)	36.52 (27.70)

© 2024 Wu Y et al. *JAMA Netw Open.*

**Weight-bearing physical activity, mean (SD), % of total physical activity**	73% (27%)	74% (27%)	72% (28%)
**Non-weight-bearing physical activity, mean (SD), % of total physical activity**	27% (27%)	26% (27%)	28% (28%)
**Weight-bearing physical activity, mean (SD), MET*Hour/week**	25.85 (23.53)	26.20 (24.60)	25.11 (21.11)
**Sports activity (i.e. Running), mean (SD), % of Weight-bearing physical activity**	18% (29%)	19% (30%)	17% (28%)
**Daily activity (i.e. Walking), mean (SD), % of weight-bearing physical activity**	82% (29%)	81% (30%)	83% (28%)
**Non-weight-bearing physical activity, mean (SD), MET*Hour/week**	11.01 (16.83)	10.82 (17.16)	11.41 (16.12)
**Sports activity (i.e. Swimming), mean (SD), % of non-weight-bearing physical activity**	22% (35%)	23% (36%)	20% (33%)
**Daily activity (i.e. Biking) , mean (SD), % of non-weight-bearing physical activity**	78% (35%)	77% (36%)	80% (33%)

*DXA= dual X-ray Absorptiometry, BMI= Body mass index, HDL= high-density lipoprotein. RS= Rotterdam study. KLG= the Kellgren and Lawrence Grade (KLG). MET*Hour/week = Hours of Metabolic equivalent of task per week. Continuous data are shown in mean with Standard deviations, and category variables are shown in the number of cases and percentage.*

© 2024 Wu Y et al. *JAMA Netw Open.*

eTable 9. Stratification analysis on tertiles of lower-limb muscle mass index (LMI) for association between physical activities and incident radiographic knee osteoarthritis in population without baseline knee pain

Population	Outcomes	Exposure	Unadjusted model		Model 1		Model 2	
			OR 95%CI	P value	OR 95%CI	P value	OR 95%CI	P value
Low Tertile：	N = 431, N knee Joints = 847, N joints with radiographic osteoarthritis incidence (%) = 27 (3.2%)	Total physical activities	1.47(1.12-1.95)	0.006*	1.66(1.22-2.26)	0.001*	1.68(1.17-2.41)	0.005*
		Non-Weight-bearing activities	0.95(0.64-1.42)	0.80	1.18(0.86-1.63)	0.30	1.19(0.87-1.63)	0.28
		Weight-bearing activities	1.52(1.14-2.03)	0.004*	1.53(1.15-2.04)	0.003*	1.52(1.08-2.14)	0.02
Middle Tertile：	N = 429, N knee Joints = 847, N joints with radiographic osteoarthritis incidence (%) = 47 (5.6%)	Total physical activities	0.76(0.47-1.23)	0.27	0.88(0.56-1.38)	0.58	0.84(0.53-1.32)	0.45
		Non-Weight-bearing activities	0.7(0.41-1.2)	0.19	0.85(0.55-1.31)	0.45	0.85(0.56-1.28)	0.44
		Weight-bearing activities	0.87(0.59-1.29)	0.49	0.93(0.63-1.38)	0.73	0.88(0.59-1.31)	0.53
High tertile	N = 413, N knee Joints = 809, N joints with radiographic osteoarthritis incidence (%) = 56 (6.9%)	Total physical activities	1(0.71-1.39)	0.99	1.06(0.76-1.48)	0.72	1.07(0.76-1.52)	0.70
		Non-Weight-bearing activities	0.9(0.71-1.13)	0.37	0.94(0.74-1.2)	0.63	0.92(0.7-1.2)	0.53
		Weight-bearing activities	1.12(0.8-1.57)	0.51	1.15(0.83-1.61)	0.40	1.2(0.84-1.7)	0.31

*The statistical model used is a multivariate logistic regression model—Model1 adjusted for age, sex, Rotterdam study sub-cohorts, BMI, follow up time, and baseline KLG. Model 2 additionally adjusted for education level, alcohol intake, smoking, systolic blood pressure, HDL/total cholesterol ratio, and diabetes mellitus. * Indicates p-value remain statistically significant after multiple testing corrections using Benjamini and Hochberg method.*

© 2024 Wu Y et al. *JAMA Netw Open.*

eTable 10. Stratification analysis on tertiles of lower-limb muscle mass index (LMI) for association between physical activities and incident radiographic knee osteoarthritis in population with baseline knee pain

Population	Outcomes	Exposure	Unadjusted model		Model 1		Model 2	
			OR 95%CI	P value	OR 95%CI	P value	OR 95%CI	P value
Low Tertile	N = 184, N knee Joints = 343, N joints with radiographic osteoarthritis incidence (%) = 30 (8.7%)	Total physical activities	0.61(0.33-1.15)	0.13	0.65(0.41-1.04)	0.08	0.74(0.41-1.34)	0.32
		Non-Weight-bearing activities	0.62(0.28-1.39)	0.25	0.66(0.3-1.43)	0.29	0.76(0.31-1.85)	0.54
		Weight-bearing activities	0.78(0.43-1.44)	0.43	0.75(0.46-1.22)	0.25	0.83(0.45-1.52)	0.54
Middle Tertile	N = 202, N knee Joints = 376, N joints with radiographic osteoarthritis incidence (%) = 41 (1.9%)	Total physical activities	0.93(0.56-1.54)	0.77	0.97(0.6-1.57)	0.92	1(0.62-1.59)	0.98
		Non-Weight-bearing activities	0.94(0.6-1.46)	0.77	0.95(0.61-1.46)	0.81	1.04(0.66-1.64)	0.86
		Weight-bearing activities	0.96(0.55-1.69)	0.90	1.02(0.61-1.71)	0.94	0.98(0.62-1.56)	0.94
High tertile	N = 222, N knee Joints = 409, N joints with radiographic osteoarthritis incidence (%) = 77 (18.8%)	Total physical activities	1.16(0.82-1.65)	0.40	1.3(0.89-1.91)	0.18	1.34(0.91-1.99)	0.14
		Non-Weight-bearing activities	0.99(0.77-1.28)	0.96	1.13(0.86-1.48)	0.39	1.15(0.87-1.52)	0.33
		Weight-bearing activities	1.2(0.84-1.71)	0.32	1.22(0.82-1.8)	0.32	1.22(0.82-1.83)	0.32

*The statistical model used is a multivariate logistic regression model—Model1 adjusted for age, sex, Rotterdam study sub-cohorts, BMI, follow up time, and baseline KLG. Model 2 additionally adjusted for education level, alcohol intake, smoking, systolic blood pressure, HDL/total cholesterol ratio, and diabetes mellitus. * Indicates p-value remain statistically significant after multiple testing corrections using Benjamini and Hochberg method.*

© 2024 Wu Y et al. *JAMA Netw Open.*

eTable 11. Association between physical activities and incident radiographic knee osteoarthritis in populations without baseline knee pain and excluding baseline KLG>=1

Population	Outcomes	Exposure	Unadjusted model		Model 1		Model 2	
			**OR 95%CI**	**P value**	**OR 95%CI**	**P value**	**OR 95%CI**	**P value**
Population with no baseline knee pain and baseline KL=0	N = 2924, N knee Joints = 5282, N joints with radiographic osteoarthritis incidence (%) = 165 (3.1%)	Total physical activities	1.1(0.94-1.3)	0.22	1.19(1.02-1.38)	0.03*	1.17(1-1.37)	0.05
		Non-Weight-bearing activities	0.89(0.74-1.08)	0.25	0.95(0.81-1.13)	0.60	0.95(0.79-1.13)	0.53
		Weight-bearing activities	1.19(1.03-1.38)	0.02	1.26(1.1-1.46)	0.001*	1.24(1.08-1.44)	0.003*

*The statistical model used is a multivariate logistic regression model—Model1 adjusted for age, sex, Rotterdam study sub-cohorts, BMI, follow up time, and baseline KLG. Model 2 additionally adjusted for education level, alcohol intake, smoking, systolic blood pressure, HDL/total cholesterol ratio, and diabetes mellitus. * Indicates p-value remain statistically significant after multiple testing corrections using Benjamini and Hochberg method.*

© 2024 Wu Y et al. *JAMA Netw Open.*

eTable 12. Association between physical activities and incident radiographic knee osteoarthritis in populations excluding baseline KLG>=1

Population	Outcomes	Exposure	Unadjusted model		Model 1		Model 2	
			OR 95%CI	P value	OR 95%CI	P value	OR 95%CI	P value
Low tertile of LMI subgroup of population with no baseline knee pain and baseline KL=0	N = 397, N knee Joints = 740, N joints with radiographic osteoarthritis incidence (%) = 18 (2.4%)	Total physical activities	1.52(1.12-2.05)	0.007*	1.67(1.2-2.33)	0.002*	1.84(1.24-2.73)	0.002*
		Non-Weight-bearing activities	1.08(0.82-1.44)	0.57	1.22(0.93-1.6)	0.16	1.29(1.01-1.64)	0.04
		Weight-bearing activities	1.52(1.12-2.06)	0.007*	1.55(1.16-2.08)	0.003*	1.65(1.19-2.3)	0.003*
Middle tertile of LMI subgroup of population with no baseline knee pain and baseline KL=0	N = 389, N knee Joints = 716, N joints with radiographic osteoarthritis incidence (%) = 28 (3.9%)	Total physical activities	0.58(0.31-1.06)	0.08	0.7(0.41-1.19)	0.19	0.67(0.39-1.16)	0.15
		Non-Weight-bearing activities	0.41(0.17-0.96)	0.04	0.51(0.24-1.07)	0.08	0.51(0.24-1.09)	0.08
		Weight-bearing activities	0.77(0.46-1.28)	0.31	0.88(0.56-1.38)	0.58	0.86(0.57-1.31)	0.49
High tertile of LMI subgroup of population with no baseline knee pain and baseline KL=0	N = 368, N knee Joints = 680, N joints with radiographic osteoarthritis incidence (%) = 33 (4.9%)	Total physical activities	1.16(0.81-1.66)	0.41	1.25(0.9-1.72)	0.18	1.24(0.88-1.75)	0.22
		Non-Weight-bearing activities	0.93(0.73-1.2)	0.59	0.96(0.76-1.22)	0.75	0.96(0.73-1.25)	0.76
		Weight-bearing activities	1.34(0.92-1.95)	0.12	1.43(1.03-1.99)	0.04	1.45(1.01-2.07)	0.05

The statistical model used is a multivariate logistic regression model—Model1 adjusted for age, sex, Rotterdam study sub-cohorts, BMI, follow up time, and baseline KLG. Model 2 additionally adjusted for education level, alcohol intake, smoking, systolic blood pressure, HDL/total cholesterol ratio, and diabetes mellitus. * *Indicates p-value remain statistically significant after multiple testing corrections using Benjamini and Hochberg method.*

© 2024 Wu Y et al. *JAMA Netw Open.*

eReferences

1. Ikram MA, Kieboom BCT, Brouwer WP, et al. The Rotterdam Study. Design update and major findings between 2020 and 2024. *Eur J Epidemiol*. Feb 7 2024;
2. Stel VS, Smit JH, Pluijm SM, Visser M, Deeg DJ, Lips P. Comparison of the LASA Physical Activity Questionnaire with a 7-day diary and pedometer. *J Clin Epidemiol*. Mar 2004;57(3):252-8.
3. Caspersen CJ, Bloemberg BPM, Saris WHM, Merritt RK, Kromhout D. The Prevalence of Selected Physical Activities and Their Relation with Coronary Heart Disease Risk Factors in Elderly Men: The Zutphen Study, 1985. *American Journal of Epidemiology*. 1991;133(11):1078-1092.
4. Koolhaas CM, Dhana K, Schoufour JD, et al. Physical activity and cause-specific mortality: the Rotterdam Study. *Int J Epidemiol*. Oct 1 2018;47(5):1705-1713.
5. Verweij LM, van Schoor NM, Dekker J, Visser M. Distinguishing four components underlying physical activity: a new approach to using physical activity questionnaire data in old age. *BMC Geriatr*. May 3 2010;10:20.
6. Van Buuren S, Groothuis-Oudshoorn K. mice: Multivariate imputation by chained equations in R. *Journal of statistical software*. 2011;45:1-67.
7. Bodner TE. What Improves with Increased Missing Data Imputations? *Struct Equ Modeling*. Oct-Dec 2008;15(4):651-675.
8. Kellgren JH, Lawrence JS. Radiological assessment of osteo-arthrosis. *Ann Rheum Dis*. Dec 1957;16(4):494-502.
9. Kerkhof HJ, Meulenbelt I, Akune T, et al. Recommendations for standardization and phenotype definitions in genetic studies of osteoarthritis: the TREAT-OA consortium. *Osteoarthritis Cartilage*. Mar 2011;19(3):254-64.
10. Benjamini Y, Hochberg Y. Controlling the False Discovery Rate - a Practical and Powerful Approach to Multiple Testing. *J R Stat Soc B*. 1995;57(1):289-300.
11. Borga M, West J, Bell JD, et al. Advanced body composition assessment: from body mass index to body composition profiling. *J Investig Med*. Jun 2018;66(5):1-9.
12. Shepherd JA, Ng BK, Sommer MJ, Heymsfield SB. Body composition by DXA. *Bone*. Nov 2017;104:101-105.
13. Tsukasaki K, Matsui Y, Arai H, et al. Association of Muscle Strength and Gait Speed with Cross-Sectional Muscle Area Determined by Mid-Thigh Computed Tomography - A Comparison with Skeletal Muscle Mass Measured by Dual-Energy X-Ray Absorptiometry. *J Frailty Aging*. 2020;9(2):82-89.

© 2024 Wu Y et al. *JAMA Netw Open.*
