# Supplementary material for: Weight-Bearing Physical Activity, Lower-Limb Muscle Mass, and Risk of Knee Osteoarthritis
Source: JAMA Netw Open. 2024 Apr 30;7(4):e248968. doi: 10.1001/jamanetworkopen.2024.8968 (PMC11061770; doi:10.1001/jamanetworkopen.2024.8968)
Supplement: Supplement 2. — Data Sharing Statement [file jamanetwopen-e248968-s002.pdf]

Data Sharing Statement

Wu. Weight-Bearing Physical Activity, Lower-Limb Muscle Mass, and Risk of Knee Osteoarthritis. *JAMA Netw Open*. Published April 30, 2024.
doi:10.1001/jamanetworkopen.2024.8968

Data
Data available: Yes
Data types: Deidentified participant data
How to access data: Data Sharing statement Data are available upon reasonable request. All relevant data supporting the key findings of this study are available within the article and its supplementary data. Due to ethical and legal restrictions (GDPR), individual-level data from the Rotterdam Study cannot be made publicly available. Data are available upon request to the data manager of the Rotterdam Study, Frank van Rooij (f.vanrooij@ erasmusmc.nl), and are subject to local rules and regulations. This includes submitting a proposal to the management team of RS, where upon approval, analysis needs to be done on a local server with protected access, complying with GDPR.
When available: With publication

Supporting Documents
Document types: None

Additional Information
Who can access the data: researchers whose proposed use of the data has been approved
Types of analyses: Dependents on the proposed analysis plan
Mechanisms of data availability: Data are available upon request to the data manager of the Rotterdam Study, Frank van Rooij (f.vanrooij@ erasmusmc.nl), and are subject to local rules and regulations. This includes submitting a proposal to the management team of RS, where upon approval, analysis needs to be done on a local server with protected access, complying with GDPR.
